# Introduction to the *RSC Advances* themed collection on nanotubes: fabrication, properties, and applications

**DOI:** 10.1039/d5ra90046h

**Published:** 2025-05-13

**Authors:** O. Durante, A. Di Bartolomeo

**Affiliations:** a Physics Department “E. R. Caianiello”, University of Salerno Fisciano Sa 84084 Italy odurante@unisa.it adibartolomeo@unisa.it

## Abstract

Ofelia Durante and Antonio Di Bartolomeo introduce the *RSC Advances* themed issue on nanotubes: fabrication, properties, and applications.
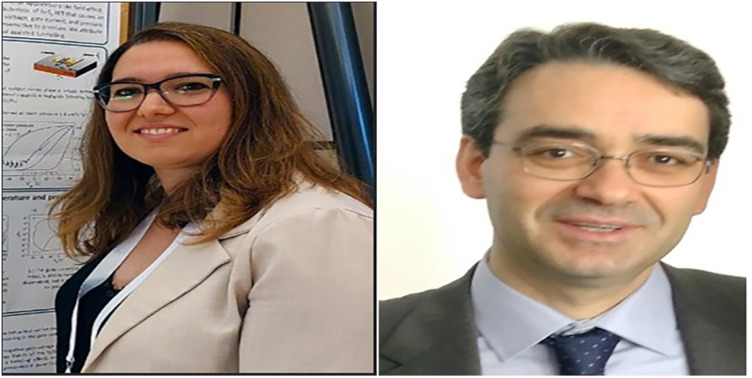

Over the past three decades, research on carbon nanotubes has led to the discovery of fundamental phenomena and has enabled the introduction of innovative applications in numerous fields, including nanoelectronics, gas sensors, photodetectors, telecommunications, quantum information, materials engineering, nanomechanics, nanofluidics, nanomedicine, pharmacology, catalysis, energy science and more.^1,2^ These successes have stimulated the exploration of nanotubes made from other materials, thereby expanding the range of applications and technological possibilities. In particular, the family of two-dimensional transition metal dichalcogenides, such as MoS_2_ and WS_2_, has opened up promising new opportunities. MoS_2_ and WS_2_ nanotubes have already been successfully synthesized and studied,^3–7^ while others, such as black phosphorus nanotubes, have been theoretically predicted but not yet experimentally confirmed. This shows how the field is constantly evolving and how new directions are always on the horizon. Research on nanotube synthesis and characterization methodologies is constantly advancing, and with it, increasingly advanced and specific applications are emerging.

This themed collection presents a selection of articles exploring new findings in the synthesis, characterization and application of nanotubes, with particular focus on carbon nanotubes and hybrid materials. These articles illustrate the current scientific challenges, discoveries and the latest applications, offering a comprehensive overview of the state of the art. Ranging from medicine to energy, from nanoelectronics to advanced sensors, the contributions demonstrate how nanotubes can have a significant impact in several fields.

Savargaonkar *et al.* (https://doi.org/10.1039/D4RA05038J) examine the use of copper-modified titanium nanotubes to enhance osteogenic differentiation of adipose tissue-derived stem cells. Research has shown that copper-modified nanotubes not only promote cell adhesion and proliferation, but also improve blood clotting properties, making them promising for applications in orthopaedic implants. This study underlines the importance of surface engineering of nanotubes for biomedical applications.

Wang *et al.* (https://doi.org/10.1039/D4RA00852A) study the crystallization of anodized TiO_2_ nanotube arrays, examining the morphological and structural evolution during annealing treatment. The results show how controlled crystallization leads to improved electrochemical properties, with potential applications in batteries and energy storage devices.

Chatree and Schulte (https://doi.org/10.1039/D3RA05688K) examine the use of carbon nanotube deposits on pencil graphite electrodes to improve analytical performance in voltammetry. This approach promises low-cost applications for advanced electroanalytical sensors, also suggesting their use in ecological biosensors and green platforms for chemical analysis.

The field of nanotubes is rapidly expanding, with a promising future for both fundamental research and practical applications. An interdisciplinary approach is crucial for progress in this field, which can be achieved through collaboration between physicists, chemists, materials scientists, life scientists, and engineers.

In conclusion, this themed collection provides an overview of the state of the art in the field of nanotubes, and opens the door to new and exciting directions for future research.

## References

[cit1] De Volder M. F. L., Tawfick S. H., Baughman R. H., Hart A. J. (2013). Science.

[cit2] Baughman R. H., Zakhidov A. A., De Heer W. A. (2002). Science.

[cit3] Pelella A., Kumar A., Intonti K., Durante O., De Stefano S., Han X., Li Z., Guo Y., Giubileo F., Camilli L., Passacantando M., Zak A., Di Bartolomeo A. (2024). Small.

[cit4] Pelella A., Camilli L., Giubileo F., Zak A., Passacantando M., Guo Y., Intonti K., Kumar A., Di Bartolomeo A. (2025). Nanoscale.

[cit5] Durante O. , Stefano S. D., Capista D., Passacantando M., Zak A., Giubileo F., Camilli L. and Sr A. D. B., in 2023 IEEE Nanotechnology Materials and Devices Conference (NMDC), IEEE, Paestum, Italy, 2023, pp. 676–680

[cit6] Grillo A., Passacantando M., Zak A., Pelella A., Di Bartolomeo A. (2020). Small.

[cit7] Remskar M., Mrzel A., Skraba Z., Jesih A., Ceh M., Demšar J., Stadelmann P., Lévy F., Mihailovic D. (2001). Science.

